# Do we treat our patients or rather periodontal microbes with adjunctive antibiotics in periodontal therapy? A 16S rDNA microbial community analysis

**DOI:** 10.1371/journal.pone.0195534

**Published:** 2018-04-18

**Authors:** Daniel Hagenfeld, Raphael Koch, Sebastian Jünemann, Karola Prior, Inga Harks, Peter Eickholz, Thomas Hoffmann, Ti-Sun Kim, Thomas Kocher, Jörg Meyle, Doğan Kaner, Ulrich Schlagenhauf, Benjamin Ehmke, Dag Harmsen

**Affiliations:** 1 Department of Periodontology and Restaurative Dentistry, Münster University Hospital, Münster, Germany; 2 Institute of Biostatistics and Clinical Research, University of Münster, Münster, Germany; 3 Center for Biotechnology – CeBiTec, University of Bielefeld, Bielefeld, Germany; 4 Department of Periodontology, Johann Wolfgang Goethe-University Frankfurt, Frankfurt, Germany; 5 Department of Periodontology, TU Dresden, Dresden, Germany; 6 Section of Periodontology, Department of Conservative Dentistry, University Hospital Heidelberg, Heidelberg, Germany; 7 Unit of Periodontology, University Medicine Greifswald, Greifswald, Germany; 8 Department of Periodontology, University of Giessen, Giessen, Germany; 9 Department of Periodontology, Dental School, Faculty of Health, University of Witten/Herdecke, Witten, Germany; 10 Departments of Periodontology and Synoptic Dentistry, Charité University Medicine Berlin, Berlin, Germany; 11 Department of Periodontology, University Hospital Würzburg, Würzburg, Germany; National Cancer Institute, UNITED STATES

## Abstract

Empiric antibiotics are often used in combination with mechanical debridement to treat patients suffering from periodontitis and to eliminate disease-associated pathogens. Until now, only a few next generation sequencing 16S rDNA amplicon based publications with rather small sample sizes studied the effect of those interventions on the subgingival microbiome. Therefore, we studied subgingival samples of 89 patients with chronic periodontitis (solely non-smokers) before and two months after therapy. Forty-seven patients received mechanical periodontal therapy only, whereas 42 patients additionally received oral administered amoxicillin plus metronidazole (500 and 400 mg, respectively; 3x/day for 7 days). Samples were sequenced with Illumina MiSeq 300 base pairs paired end technology (V3 and V4 hypervariable regions of the 16S rDNA). Inter-group differences before and after therapy of clinical variables (percentage of sites with pocket depth ≥ 5mm, percentage of sites with bleeding on probing) and microbiome variables (diversity, richness, evenness, and dissimilarity) were calculated, a principal coordinate analysis (PCoA) was conducted, and differential abundance of agglomerated ribosomal sequence variants (aRSVs) classified on genus level was calculated using a negative binomial regression model. We found statistically noticeable decreased richness, and increased dissimilarity in the antibiotic, but not in the placebo group after therapy. The PCoA revealed a clear compositional separation of microbiomes after therapy in the antibiotic group, which could not be seen in the group receiving mechanical therapy only. This difference was even more pronounced on aRSV level. Here, adjunctive antibiotics were able to induce a microbiome shift by statistically noticeably reducing aRSVs belonging to genera containing disease-associated species, *e*.*g*., *Porphyromonas*, *Tannerella*, *Treponema*, and *Aggregatibacter*, and by noticeably increasing genera containing health-associated species. Mechanical therapy alone did not statistically noticeably affect any disease-associated taxa. Despite the difference in microbiome modulation both therapies improved the tested clinical parameters after two months. These results cast doubt on the relevance of the elimination and/or reduction of disease-associated taxa as a main goal of periodontal therapy.

## Introduction

The oral cavity provides a unique eco-system with different niches for microbial organisms harboring a diverse microbiota with approximately 700 different prokaryote species [1]. These microbes live in the human hosts and play important roles in health and disease. A mutual dependence exists between the host and the microbiome. Perturbations in this equilibrium can lead to a dysbiosis and consequently to a diseased host [2]. In case of the periodontium a dysbiosis of the complex subgingival microbiome leads to the formation of periodontal pockets and destruction of tooth supporting tissue in most cases without notification of the host. Approximately 50% of the adult population suffer from moderate or severe forms of periodontitis in the United States [3].

Previous research on periodontal therapy focused on identifying bacterial species within the subgingival microbiome that are associated with periodontitis and to develop strategies to eradicate these pathogens. In a fundamental work by the group of Socransky and Haffajee [4] a microbial succession was postulated describing green, yellow and purple complex bacteria associated with periodontal health, to an orange and ultimately a red complex with pathogens, *i*.*e*., *Porphyromonas ginigvalis*, *Treponema denticola*, and *Tannerella forsythia* frequently found in periodontitis. Reduction of these pathogens after mechanical therapy can be improved by adjunctive systemic antibiotics, *e*.*g*., amoxicillin and metronidazole and even elimination of other key-pathogens as *Aggregatibacter actinomycetemcomitans* can be achieved [5,6]. For decades dentists used empiric antibiotics to treat periodontitis by eliminating those periodontal pathogens although strong clinical evidence is missing [7]. A recent large multi-center randomized controlled trial (ABPARO study) showed that the additional benefit of empiric adjunctive antibiotics was of questionable clinical relevance [8,9].

Next generation sequencing (NGS) greatly expanded periodontal microbial community analyses because it is not limited to cultivated pathogens or pre-selection of targeted species and allows for a deeper understanding of the whole microbiome dynamics after periodontal therapy [10]. By using 16S ribosomal DNA (rDNA) sequence analysis we documented that not only pathogenic taxa were eliminated in the antibiotics group, but the entire microbiome shifted after therapy [11]. However, in this study only 4 patients of the ABPARO study were investigated using the Ion Torrent Personal Genome Machine (PGM), which had limited read length and a relative high homopolymer related sequencing error rate [12]. Other amplicon NGS studies on periodontal therapy using the 454-pyrosequencing technology with similar homopolymer error rates had also rather limited sample sizes [13–15] and did not include an adjunctive antibiotic group [13,14].

The aim of this study was to explore the short-term effects of mechanical periodontal therapy with or without adjunctive amoxicillin and metronidazole on the subgingival microbiome using Illumina MiSeq sequencing technology in a large subgroup of non-smoking patients from the ABPARO study.

## Material and methods

### Patient cohort and sampling procedure

Specimens from the ABPARO study—a multicenter randomized, double-blinded, parallel group, and placebo-controlled study—were used (ISRCTN: 64254080, Clinical Trials.gov NCT00707369). Here, patients with untreated chronic periodontitis (localized: <30% and generalized: ≥30% of teeth with moderate: ≥3mm to <5mm and severe: ≥5mm attachment loss [16]) were recruited, who received mechanical debridement plus 500 mg amoxicillin and 400 mg metronidazole three times daily for 7 days (antibiotic) or mechanical debridement and placebo (placebo). All mechanical debridement was performed in up to two sessions on two consecutive days with hand instruments and/or machine-driven scalers. Supportive periodontal therapy was performed in three months intervals over a 24-months period [17].

For this study, 96 non-smoking patients (CO-level in exhaled air <7 ppm) from the per-protocol collective of the ABPARO study (270 patients; 11.5% with localized severe, 51.1% with localized severe and generalized moderate, and 37.4% with generalized severe chronic periodontitis) were chosen [8]. Patients were selected randomly with respect to represent the observed allocation of therapy groups, gender, and severity of periodontitis in the ABPARO study. Patients of this selection had to be anonymized again and only subgingival samples taken before and two months after intervention (short-term) were analyzed here [18].

Subgingival specimens for microbiological analysis were taken from four teeth with a pocket probing depth of ≥6 mm, one in each quadrant. Detailed teeth selection has been described previously [19]. At the four sample sites supragingival plaque was gently removed, teeth were air-dried and isolated with cotton rolls. One sterile paper point (ISO45, Roeko Dental, Langenau, Germany) was inserted for 10 seconds in each site and all paper points were removed and pooled in one sterile collection tube. Samples were stored at −20°C until further use. The study database was anonymized. This study was approved by the Medical Ethics Committee of the University of Muenster (ref: 2016-505-f-S).

### DNA extraction, 16S rDNA amplification, and amplicon sequencing

Bacterial genomic DNA was isolated and purified with the QiaAmp Mini DNA-Isolation Kit (Qiagen, Hilden, Germany). The protocol followed the manufacturer’s instructions with minor modifications, *i*.*e*., i) after addition of lysis buffer ATL samples were sonicated in an ultrasonic water bath (Sonotex RK 82, Bandelin Electronic AG, Berlin, Germany) for 5 min to elute the collected plaque from the paperpoints, and ii) the pretreatment step with proteinase K (20 mg/ml) at 56 °C was carried out overnight. The purified DNA was eluted in 200 μl of elution buffer. DNA concentration was estimated with the Qubit 2.0 instrument applying the Qubit dsDNA HS Assay (Life Technologies, Invitrogen division, Darmstadt, Germany). For NGS library preparation, the recommended protocol for preparing 16S ribosomal RNA gene amplicons for the Illumina MiSeq system was used [20]. The suggested universal bacterial primers were utilized for amplifying the V3 and V4 hypervariable regions of the bacterial 16S rRNA gene with polymerase chain reaction (PCR) using the KAPA Hifi HotStart Ready Mix (Roche Diagnostics Deutschland, Mannheim, Germany). Purity and exact fragment size of amplicons was determined with the Caliper GX system using the HT DNA High Sensitivity LabChip Kit (PerkinElmer, Rodgau, Germany). In a second PCR sample-specific “barcode”-primers and adapter sequences were attached. Up to 96 libraries were normalized and pooled for an Illumina MiSeq sequencing run using the MiSeq Reagent Kit version (v.) 3 with marginally overlapping 300 base pairs (bp) paired end reads.

### 16S rDNA sequence processing

MiSeq paired end reads were screened for matching forward and reverse amplification primers (forward primer 5’-CCTACGGGNGGCWGCAG-3’, reverse primer 5’-GACTACHVGGGTATCTAATCC-3’), using Cutadapt v.1.8.1 [21] with anchor flags ('*-g^*' for forward and '*-G^'* for reverse primer), a minimum overlap of ten bases, and a maximum error rate of 0.2. Matched primers were trimmed and reads that did not contain the adapter sequence or where it could not be identified were completely removed from the output file. Primer trimmed reads were submitted to the European Nucleotide Archive (http://www.ebi.ac.uk/ena/) of EMBL European Bioinformatics Institute under the study accession number PRJEB18651.

Raw reads were then processed using the R language environment v.3.4.3 [22] and RStudio v.1.0.153 [23], following the DADA2 workflow described by Callahan et al. [24]. Reads were truncated (forward reads at position 260 and reverse reads at position 195 onwards) and filtered (maximum of 2 expected errors per read) on paired ends jointly. Reads were de-replicated and combined with the corresponding abundance and a summary of the quality information associated with this sequence pattern. Sequence variants in each sample were inferred using the high-resolution DADA2 method, which relies on a parameterized model of substitution errors to distinguish sequencing errors from real biological variation [25]. Denoised forward and reverse reads were then merged requiring at least 15 bp overlap and chimeras were subsequently removed from the data set. To those ribosomal sequence variants (RSV) taxonomic labels were assigned with a naive Bayesian classifier using the Silva v.128 training set [26]. For quality control reasons rarefaction curves were generated for each sample individually. Samples below 10,000 total reads and/or with a rarefaction curve that did not reach a plateau phase were rejected.

After multiple alignment of RSVs with the command *decipher*::*AlignSeqs* from the R-package DECIPHER [27] a neighbor-joining tree [28] was created using the phangorn package [29]. Utilizing the R-package phyloseq v.1.19.1 [30] the following sample specific details were combined: I) all non-chimeric RSVs along with their classification down to genus-level and their abundance (S1 Table); ii) the phylogenetic tree; and iii) the patient identifier, treatment group (antibiotics or placebo), and treatment time point (before or after therapy; S2 Table). To remove spurious RSVs all variants occurring in two or less samples were removed from the data set with *phyloseq*::*prune_taxa*. To compensate for possible sequencing errors closely-related RSVs were tree-based agglomerated using single-linkage clustering. Thereby, all tips of the phylogenetic tree, which were separated by a cophenetic distance smaller than h = 0.03, were agglomerated with *phyloseq*::*tip_glom*. Those agglomerated RSVs are designated as aRSVs hereinafter.

### Statistical analysis of clinical and microbial variables

All inferential statistics were intended to be exploratory instead of confirmatory. P-values were considered statistically noticeable if p≤0.05. Statistical analysis of demographic and clinical variables was performed using the SAS System for Windows (SAS Institute, Cary, NC, USA). Available demographic variables were age and gender. For clinical variables, the proportion of tooth sites per patient with periodontal pocket depth ≥ 5 mm (%PPD5mm), the proportion of tooth sites per patient with further relative attachment loss after baseline ≥ 1.3 mm (%RAL1.3mm), and the proportion of tooth sites per patient with bleeding on probing (%BOP) were used. Continuous variables were reported as mean ± standard deviation and median (25% quantile, 75% quantile). Differences between antibiotic and placebo group were tested using Fisher’s exact test for categorical variables, *i*.*e*., age and gender, and two-sided Mann-Whitney U tests for all continuous variables. Wilcoxon signed-rank tests were performed to analyze the change of continuous variables between before and after treatment.

Microbial variables were analyzed using the R-package phyloseq. To visualize the distribution of read counts per aRSV over all samples a bar plot was created (data not shown). To allow for comparison of alpha diversity measurements between samples, reads were randomly sub-sampled to the level of the sample with the least number of reads, *i*.*e*., 11,763, with the command *phyloseq*::*rarefy_even_depth*. For measurement of richness, the number of observed aRSVs and for diversity the Shannon index in each sample was determined [31] by using the command *phyloseq*::*estimate_richness*. The Pielou index was used to measure community evenness, *i*.*e*., dividing the Shannon index by the natural logarithm of observed aRSVs for each sample [32]. For beta-diversity a Bray-Curtis distance matrix was created with the command *phyloseq*::*distance*. All diversity indices and their changes were compared between both treatment groups by applying Mann-Whitney U tests. Wilcoxon signed-rank tests were performed to analyze the change of each diversity index between before and after treatment in the respective treatment group. To explore community structure and reduce dimensionality, a principal coordinates analysis (PCoA) was done with the Bray-Curtis dissimilarity matrix by eliciting the commands: *phyloseq*::*ordinate* and *phyloseq*::*plot_ordination*.

The analysis of differential abundance of aRSVs was done with the R-package DESeq2 v.1.18.1 [33] using non-normalized aRSV read counts as input with the command *deseq2*::*phyloseq_to_deseq2*. This package uses a negative binomial distribution and accounts for overdispersion with varying aRSV read counts. The overdispersion parameter is estimated by comparing the mean aRSV-abundance over all samples. As part of the DESeq2 model the default independent filtering was used to increase the detection rate of differentially abundant aRSVs after false discovery correction [31,33]. To account for dependencies between multiple samples within one patient, it was first tried to specify a model including the patient as a fixed effect. Because of only two sampling time points per patient, before and after therapy, and too many patient parameters to be estimated, this model was over-specified and could not be fitted. To the best of our knowledge, for the DESeq2 package and other common R-packages the option to include a random effect for the patient is not possible. So we decided to regard the repeated samples within each patient as independent, which might lead to biased variance estimates of the log2-fold regression parameters [33,34]. The fitted design formula of this second model was used for further calculations. It contained the main effects of treatment group and time point, and the interaction between both variables as fixed effects. The *betaPrior* parameter was set to false and a local dispersion fit was selected. The false discovery rate was set to 0.05 and controlled by applying the Benjamini-Hochberg procedure to adjust the p-values [35]. Effects on the counts of an aRSV were considered as noticeable if adjusted p-value (p_adj_) ≤0.05. Noticeably differential abundant aRSVs were taxonomically labeled on genus level when possible. If such genera included species previously described by Socransky *et al*. [4], this genus was allocated to the given complex. The two genera harboring species present in more than one complex, namely, *Streptococcus* (one orange and six yellow complex associated species) and *Campylobacter* (one green and three orange complex associated species) were allocated to the complex of higher conformity (*Streptococcus* to the yellow and *Campylobacter* to the orange complex, respectively). All figures were created with the R-package ggplot2 [36].

## Results

Out of 192 samples initially processed from patients suffering from chronic periodontitis 24 samples did not satisfy quality criteria. These samples were re-introduced into an additional sequencing run. After repetition, still 6 post-treatment samples and 1 pre-treatment sample showed an insufficient quality and were thus removed from further analysis. To maintain pairwise comparisons, the related 7 paired samples were also excluded from further analysis, so that 178 samples from 47 placebo and 42 antibiotic patients remained for statistical analysis. There were no noticeable demographic differences between the two treatment groups in the categories age (1) <45years (y), (2) ≥45y and <55y, (3) ≥55y (placebo: (1) n = 8, (2) n = 14, (3) n = 25; antibiotics: (1) n = 4, (2) n = 16, (3) n = 22; p = 0.507) and gender (placebo: n = 23 females (55%), and antibiotics: n = 19 females (45%); p = 0.832). Moreover, no noticeable differences of the clinical variables %PPD5mm (p = 0.164) and %BOP (p = 0.083) could be observed between both groups before therapy. Two months after periodontal therapy clinical parameters %PPD5mm and %BOP showed a decreased proportion in both groups, but were not noticeably different between the antibiotic and placebo group (p = 0.194 and p = 0.101, respectively) (Table 1). After therapy, the proportion of further relative attachment loss (%RAL1.3mm) since baseline was also not statistically noticeably different between the antibiotic and placebo group (p = 0.752).

**Table 1:** Clinical and microbial variables for the placebo and antibiotic group at baseline and 2 months after therapy.

	Placebo (n = 47)			Antibiotic (n = 42)		
	Baseline	2 months after therapy	P-value	Baseline	2 months after therapy	P-value
**Clinical variables**						
**%PPD5mm**^#^	17.9 ± 12.0	10.4± 9.5	**<0.001**	20.9 ± 14.0	7.7 ± 6.9	**<0.001**
	14 (11, 21)	8 (5, 13)		19 (12, 25)	6 (2, 13)	
**%BOP**^#^	34.4 ± 18.6	18.0 ± 13.2	**<0.001**	41.5 ± 22.8	13.7 ± 11.9	**<0.001**
	27 (20, 52)	15 (7, 27)		37 (28, 58)	10 (5, 20)	
**%RAL1.3mm**^#^		3.8 ± 4.1			3.9 ± 5.0	
		2 (1, 6)			2 (1, 5)	
**Microbiome variables**						
**Richness**	103.21 ± 40.57	98.80 ± 41.93	0.122	108.49 ± 30.85	83.89 ± 29.69	**<0.001**
	99 (86, 128)	94 (68, 126)		112 (85, 131)	81 (63, 103)	
**Evenness**^#^	0.73 ± 0.07	0.73 ± 0.07	0.863	0.72 ± 0.05	0.73 ± 0.08	0.214
	0.75 (0.69, 0.78)	0.75 (0.71, 0.78)		0.73 (0.69, 0.75)	0.74 (0.70, 0.77)	
**Diversity**^#^	3.32 ± 0.54	3.30 ± 0.56	0.822	3.34 ± 0.41	3.20 ± 0.51	0.057
	3.33 (3.02, 3.67)	3.30 (2.99, 3.67)		3.45 (3.12, 3.61)	3.30 (3.02, 3.50)	
**Dissimilarity**^#^	0.69 ± 0.05	0.70 ± 0.05	0.193	0.69 ± 0.05	0.74 ± 0.05	**<0.001**
	0.69 (0.66, 0.72)	0.69 (0.67, 0.73)		0.67 (0.66, 0.71)	0.73 (0.71, 0.75)	

%PPD5mm, percentage of tooth sites with pocket depth ≥5 mm; %BOP, percentage of tooth sites with bleeding on probing; %RAL1.3mm, percentage of tooth sites with further relative attachment loss of ≥1.3mm between baseline and 2 months after therapy; Richness, number of aRSVs; Evenness, Pielou index; Diversity, Shannon-index; Dissimilarity, Bray-Curtis index. All variables are shown as mean ± standard deviation and median (25% quantile, 75% quantile). P-values are derived from Wilcoxon signed-rank tests comparing the variables between before and after treatment within each group.

^#^ Skewed distributed variables.

After processing the raw reads with DADA2 we found 6,596 non-chimeric unique RSVs over all samples. By removing all RSVs occurring in two or less samples this number was reduced to 1,964 RSVs. Tree-based agglomeration of the remaining RSVs resulted in 379 aRSVs, which included 17 aRSVs belonging to eukaryotes and 2 aRSVs without taxonomic assignment on kingdom level. For further analysis only the 360 bacterial aRSVs were selected that were categorized into 15 uniquely named taxa on phylum level and 120 on genus level. Read counts per aRSV were logarithmically distributed, with only few high abundant and many low abundant aRSVs (S3 Table). No differences between richness (p = 0.401), evenness (p = 0.217), diversity (p = 0.887), and dissimilarity (p = 0.444) could be observed between both groups before therapy. The most frequently found genera were *Fusobacterium* with 16.44% mean relative read count before therapy (MRRC_b_) over both groups, *Porphyromonas* (9.52% MRRC_b_), *Tanerella* (5.08% MRRC_b_), and *Fretibacterium* (3.33% MRRC_b_). For inter-sample comparisons of alpha diversity only, reads were randomly sub-sampled to the level of the sample with the least number of reads, which reduced the mean number of aRSVs over all samples from 101.9 to 98.7 aRSVs. To describe possible changes in richness of the microbial community, the number of observed aRSVs in each sample was counted (Table 1). Only in the antibiotic group richness decreased statistically noticeable after therapy (p<0.001). To investigate general changes in aRSV abundances, the Pielou and the Shannon index were calculated. The Pielou index measures the evenness of aRSV abundances with a maximum value of 1 if all aRSV abundances are equally distributed. Evenness did not change noticeably in both groups. The Shannon index, a diversity metric that takes both richness and evenness of the microbial distribution into account, did neither change noticeably in the placebo nor in the antibiotic group after therapy. The Bray-Curtis dissimilarity measures the degree of difference between microbiomes. It is low, if microbiomes show a high aRSV conformity (minimum 0) and high if microbiomes are highly dissimilar to each other (maximum 1). After periodontal therapy dissimilarity increased in both groups, but only in the antibiotic group this increase was statistically noticeable (p<0.001).

**Fig 1:**
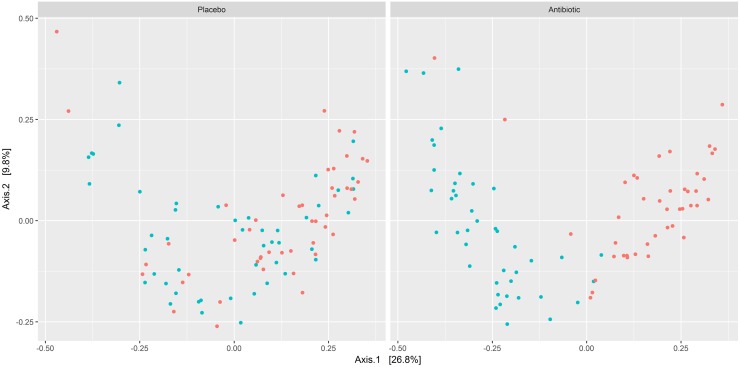
PCoA scatterplots of Bray-Curtis dissimilarities for placebo and antibiotic samples before and after periodontal therapy. For each treatment modality samples are visualized by dots, which are colored red when taken before or blue when taken after therapy. The ordination was constructed using a Bray-Curtis distance matrix. Principal component 1 (Axis 1) and principal component 2 (Axis 2) are plotted on the x- and y-axes, respectively. The percentage of variation explained by the plotted principal coordinates is indicated on the axes.

A PCoA was performed to ordinate multivariate microbiome dissimilarities based on a Bray-Curtis matrix. Ordination techniques, such as PCoA, reduce the dimensionality of microbiome data sets so that a summary of the beta diversity relationships can be visualized in scatterplots. Long distances between dots in the scatterplot visualize a high dissimilarity of microbiomes, whereas microbiomes with a similar composition are clustered together. In the antibiotic group a clear separation of microbiomes before and after treatment could be observed whereas in the placebo group such a distinct separation was not detected (Fig 1). 26.8% and 9.8% of the total variance found in the data set could be explained by the first and second axis of the PCoA scatterplots, respectively.

**Fig 2:**
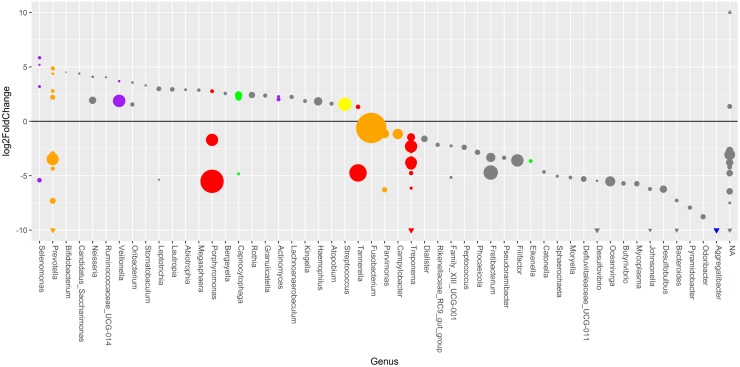
Bubble chart of aRSV abundance changes after periodontal therapy classified on genus level for the antibiotic group based on a negative binomial regression model. Bubbles represent 110 aRSVs belonging to 52 uniquely named genera (x-axis) that showed statistically noticeably changes (y-axis) in the antibiotic group after therapy on a log2-scale. All aRSVs unclassified on genus level are grouped together in an unclassified genus bin (NA). The sizes of the bubbles represent the mean relative aRSV abundance over all samples before therapy. Those aRSVs with ≥ 10 log2fold and ≤ -10 log2fold changes were marked as triangles on their respective y-axis section. All aRSVs belonging to a genus that includes species previously described by Socransky and colleagues [4] are colored according to their complex affiliation.

To describe abundance changes for single microbial taxa (aRSV and genus level, respectively) the DESeq2 package was used that does not need to sub-sample reads, thus allowing for the consideration of all identified aRSVs [31]. Only 1 aRSV found in total were noticeably differently abundant between placebo and antibiotic group before therapy (data not shown). This aRSV, belonged to a low abundant unclassified genus with 0.04% MRRC_b_, and was taxonomically labeled on phylum level as *Saccharibacteria*. After therapy, the number of differently abundant aRSVs increased to 110. In the placebo group only 3 aRSVs were noticeably differently abundant after therapy that also changed in the antibiotic group. In the antibiotic group 40 aRSVs were noticeably higher and 70 aRSVs were noticeably lower abundant after therapy (Fig 2). Fourteen of the 110 differently abundant aRSVs after therapy (all belonging to the antibiotic group) remained unclassified on genus level.

The 19 high-abundant aRSVs with a minimal baseline abundance of 1% MRRC_b_ represented 55% of all reads. In the placebo group MRRC_b_ values of 14 of high-abundant aRSVs decreased and 5 increased, but none were statistically noticeably differently abundant after therapy (S3 Table). In the antibiotic group 17 of the high abundant aRSVs showed a statistically noticeably different abundance after therapy (only 2 aRSVs taxonomically labeled as *Selenomonas* and *Prevotella* exhibited no noticeable difference). Of those 17 high-abundant aRSVs, 15 decreased and 2 increased after antibiotic therapy. Ten of the decreasing aRSVs in the antibiotic group belonged to genera containing species associated with periodontitis (red-complex: 3 aRSVs from the genus *Treponema*, 2 aRSVs *Porphyromonas*, 1 aRSV *Tannerella*; orange-complex: 1 aRSV each from *Prevotella*, *Campylobacter*, *Fusobacterium*, *Parvimonas*) as described by Socransky and colleagues. Four noticeably decreased aRSVs not allocated to a Socransky complex belonged to the genera: *Fretibacterium* (2 aRSVs with MRRC_b_ 3.33%, 1.22%), *Filifactor* (MRRC_b_ 2.39%), and *Oceanivirga* (MRRC_b_ 1.53%). Finally, one noticeably decreased aRSV was unclassified on genus level (family: *Porphyromonadaceae*, MRRC_b_ 1.75%). The two high-abundant and noticeably increasing aRSVs were assigned to genera belonging to the yellow and purple Socransky complexes associated with periodontal health, *i*.*e*., *Streptococcus* (MRRC_b_ 2.79%) and *Veillonella* (MRRC_b_ 2.44%).

The 341 low abundant aRSVs (<1% of MRRC_b_) represented 95% of all classified aRSVs, but comprised 45% of the total reads, only. Of those aRSVs, 3 changed statistically noticeably in the placebo and 93 in the antibiotic group. All of the aRSVs that noticeably changed in the placebo group also changed noticeably in the antibiotic group, *i*.*e*., *Rothia* (MRRC_b_ 0.49%; 2.22 log2fold), *Pyramidobacter* (MRRC_b_ 0.18%; -20.72 log2fold), and *Kingella* (MRRC_b_ 0.12%; 2.89 log2fold). In the antibiotic group 38 aRSVs with a low abundance at baseline noticeably increased and 55 noticeably decreased after therapy. Roughly half of the aRSVs (16/38), which noticeably increased in the antibiotic group could be associated to a Socransky complex. Most of those aRSVs are linked to periodontal health, *i*.*e*., *Capnocytophaga* (green), *Actinomyces*, *Veillonella*, and *Selenomonas* (purple). However, also some aRSVs of *Porphyromonas* and *Tannerella* from the red complex and *Campylobacter* and *Prevotella* from the orange complex noticeably increased in the antibiotic group. The 22 noticeably increasing aRSVs that could not be allocated to a Socransky complex belonged mostly to gram-positive phyla like *Firmicutes* and *Actinobacteria*. For the 55 noticeably decreasing low abundant aRSVs in the antibiotic group about one third could be assigned to a Socransky complex (21/55). Here several aRSVs belonging to the genus *Treponema* of the red and *Prevotella* of the orange complex decreased noticeably. Also, of note is a single aRSV identified as member of the genus *Aggregatibacter* that showed a fairly strong noticeable decrease of -25.34 log2-fold and on case level, even got eradicated after antibiotic therapy. Furthermore, aRSVs belonging to the genera *Capnocytophaga* and *Eikenella* associated with the green complex and *Selenomonas* from the purple complex decreased noticeably. The remaining two third of low abundant aRSVs were not assigned to a Socransky complex (34/55). This group consists mostly of aRSVs belonging to the phyla *Firmicutes* (19 aRSVs), *Bacteriodetes* (5 aRSVs), and *Proteobacteria* (5 aRSVs).

## Discussion

In this study we used the Illumina MiSeq technology with paired end 300 bp sequencing reads to analyze the subgingival microbiomes of 89 untreated chronic periodontitis patients before and 2 months after periodontal therapy with or without antibiotic use. Two months after therapy the subgingival microbiome shifted noticeably to a decreased richness and an increased dissimilarity in the antibiotic, but not in the placebo group (Table 1). The PCoA analysis revealed a general compositional separation of microbiomes in the antibiotic group and an absence of such a clear compositional separation in the placebo group (Fig 1). Differences between both treatment groups after therapy became even more apparent on genus and aRSV level (Fig 2) demonstrating the influence of adjunctive antibiotics in initiating a shift of the subgingival periodontal microbiome composition. In contrast, the placebo group therapy did not uniformly affect specific taxa to a statistically noticeable degree, with the exception of only few and low abundant aRSVs. An improvement of clinical variables was noted in both groups after therapy independently of the difference in microbial changes.

In our microbial community analysis, we observed overall 120 unique genera and detected on average 103.21 ± 40.57 aRSVs per sample in the placebo and 108.49 ± 30.85 aRSVs per sample in the antibiotic group before therapy. Compared to our present study other groups examining subjects with aggressive [13], as well as chronic periodontitis [37] reported similar numbers of aRSVs per sample. Additionally, our Shannon and Pielou indices at baseline are in concordance with other studies focusing on subjects with chronic periodontitis [11,15,37–39].

We found a statistically noticeable decrease in richness only in the antibiotic group after therapy. However in a metagenomic study of chronic periodontitis patients a decreased richness after mechanical periodontal therapy without antibiotics was observed [40]. We found no noticeable changes in the Pielou and Shannon indices after therapy in both groups, which is in concordance with several previous studies including both groups, antibiotic and placebo [15], and with placebo only [13,14]. We found an increased dissimilarity and a clear compositional separation in the PCoA after therapy in the antibiotic group, only. However, a statistically significantly increased dissimilarity and a compositional separation was reported previously in both groups after therapy [15].

Compositional differences between both treatment groups after therapy became even more apparent on genus and aRSV level. In the antibiotic group the abundance of the most prevalent aRSVs at baseline was reduced after therapy, especially for many annotated genera including species from the red and orange Socransky complexes. Furthermore, usage of antibiotics increased the number of aRSVs assigned to health-associated green, purple, and yellow complex taxa. On the contrary, in the placebo group the abundance of highly prevalent taxa belonging to the red or orange Socransky complex was not noticeably changed. This is in concordance with results of smaller microbiome studies that found no significant changes on taxa level after mechanical periodontal therapy alone [13,14]. However, in the metagenomic shotgun sequencing analysis by Shi *et al*. [40] several disease-associated taxa were significantly reduced after mechanical periodontal therapy alone. Of note in this study, only selected periodontal pockets that clinically improved after treatment were included in the analysis. This sampling approach differs markedly from ours. These results may show either the influence of changes of the habitat due to the decreasing pocket depth or a microbiome switch that consequently resulted in a resolved pocket. In the study by Bizzarro *et al*. the taxa level analysis showed that the therapy resulted in a significantly stronger decrease of *Porphyromonas*, *Treponema*, and *Synergistaceae* after 3 months in the antibiotic compared to the placebo group. Additionally, 3 other genera were reduced in the antibiotic group exclusively, *i*.*e*., *Paludibacter*, *Fusobacterium*, and *Parvimonas*. Furthermore, *Neisseria*, *Rothia*, *Capnocytophaga*, and *Streptococcus* increased in both groups, while *Veillonella* and *Haemophilus* increased significantly only after antibiotic exposure [15]. We found also a stronger reduction of those described taxa in the antibiotic group. However, the number of significantly differentially abundant taxa after mechanical treatment without antibiotics reported in that study differs from our findings and also from other microbiome studies [13,14]. Bizzarro *et al*. subsampled at 1400 reads per sample, analyzed only the 21 major genera, and—most importantly—did not correct for multiple testing that might explain the differences to our observations in the placebo group. Other studies analyzing selected bacteria using, *e*.*g*., culture [41] or checkerboard DNA-DNA hybridization [42] also reported a statistically significant reduction of putative pathogens in the placebo group, which was stronger in the antibiotic group. Our results did also show a reduction of the mean relative abundances of putative periodontal pathogens in the placebo group, which was, however, not statistically noticeable. Of note, patients included in those studies that showed also better clinical results in the antibiotic group, had a much higher proportion of deep periodontal pockets ≥5 mm at baseline compared to our study (approximately 38/44% vs. 19% PPD5mm).

In contrast to all other cited studies we found a larger number of low abundant aRSVs noticeably different after therapy, in the antibiotic group. We used DESeq2 for aRSV level analysis that increased the detection rate for single aRSVs, because microbiomes were not needed to be rarefied for this analysis [43]. DESeq2 performed in the past favorably compared to other tools for identification of differentially expressed genes [34] and has also successfully been used with taxa abundance data [44]. Taken together, the use of modern sequencing techniques in principal allows for detection of low abundant taxa beyond the Socransky complex species [45,46]. Indeed, approximately two third of the decreased low abundant taxa after therapy in the antibiotic group did not belong to the classical periodontal complexes.

There are some limitations in our study that warrant further comments. The examined patients had a rather low proportion of deep periodontal pockets (approximately 19% PPD5mm at baseline). A higher taxonomic resolution to species level would be desirable, but due to the limited read length of our currently used technology this was not feasible. It has been shown previously that the choice of the amplified 16S rDNA region (*e*.*g*., V3-V4 vs. V4) may influence the number of erroneous sequences using Illumina MiSeq sequencing [47]. DESeq2 does not allow to include random effects, *e*.*g*., to account for dependencies between multiple samples from the same patient. Furthermore, a whole-genome shotgun analysis would provide also insights in the functional capacities of the microbiomes. Additionally, a longer follow up would be needed to analyze the stability of the detected microbiome shift.

In summary, adjunctive antibiotics were able to induce a microbiome shift by statistically noticeably reducing disease-associated taxa and increasing health-associated taxa two month after therapy. Mechanical therapy with placebos did not induce a statistically noticeable microbiome shift. Despite the difference in microbial changes both therapies noticeably improved the tested clinical outcome parameters. This casts doubt on the relevance of the short-term (two-months) reduction and/or elimination of disease-associated taxa as a main goal of periodontal therapy.

## Supporting information

S1 Table aRSV-ID, taxonomic classifications from phylum to genus level, read counts for each sample-ID, and sequence per aRSV (n = 360). (XLSX)

S2 Table Sample-ID, anonymized patient identifier (patient-ID), treatment time point (before or after therapy), treatment group (antibiotic or placebo group), and ENA accession no. per sample (n = 178). (XLSX)

S3 Table OUT-ID, log2FoldChange, standard error of log2FoldChange (lfcSE), p-value, adjusted p-values (padj), mean relative read counts (MRRC_b_; over both groups), MRRC (before/placebo or antibiotic), MRRC (after/placebo or antibiotic), phylum, and genus (n = 360). MRRC variables are shown as mean percentages of aRSVs per sample for the respective treatment group and time point. Adjusted p-values (according to Benjamini-Hochberg) are derived from a negative binomial regression model comparing aRSV abundances before and after treatment within each group. Noticeable adjusted p-values (p_adj_ <0.05) are shown in bold. Taxonomic classifications are colored according to the Soccransky *et al*. complex affiliation. For each treatment group (Antibiotic or Placebo group) a separate sheet is used in the file. Unclassified = no taxonomic classification at this level possible; NA = not available, p_adj_ filtered out due to default independent filtering of DESeq2.
(XLSX)
